# Elevated serum levels of bone morphogenetic protein-9 are associated with better outcome in AQP4-IgG seropositive NMOSD

**DOI:** 10.1038/s41598-023-30594-z

**Published:** 2023-03-02

**Authors:** Hiroki Masuda, Masahiro Mori, Akiyuki Uzawa, Tomohiko Uchida, Mayumi Muto, Ryohei Ohtani, Reiji Aoki, Satoshi Kuwabara

**Affiliations:** 1 Department of Neurology, Graduate School of Medicine, Chiba University, 1-8-1, Inohana, Chuo-Ku, Chiba-Shi, 260-8670 Japan; 2 Department of Neurology, Chiba Rosai Hospital, 2-16, Tatsumidai-Higashi, Ichihara-Shi, 290-0003 Japan; 3 Department of Neurology, Kimitsu Chuo Hospital, 1010, Sakurai, Kisarazu-Shi, 292-8535 Japan

**Keywords:** Cytokines, Neuroimmunology, Immunology, Neurology, Neurological disorders

## Abstract

Lymphatic drainage in the central nervous system is regulated by meningeal lymphatic vasculature, and recurrent neuroinflammation alters lymphatic vessel remodeling. Patients with aquaporin-4 antibody-positive neuromyelitis optica spectrum disorder (AQP4 + NMOSD) were reported to demonstrate worse outcomes compared with patients with anti-myelin oligodendrocyte glycoprotein-associated disorders (MOGAD). This study aimed to investigate the serum cytokines relevant to vascular remodeling after attacks and their prognostic role in patients with AQP4 + NMOSD. This study measured the serum levels of 12 cytokines relevant to vascular remodeling, including bone morphogenetic protein-9 (BMP-9) and leptin, in 20 patients with AQP4 + NMOSD and 17 healthy controls (HCs). Disease controls included 18 patients with MOGAD. Serum and cerebrospinal fluid interleukin-6 levels were also measured. Clinical severity was evaluated with Kurtzke’s Expanded Disability Status Scale (EDSS). Compared with HCs, patients with AQP4 + NMOSD showed higher BMP-9 (median; 127 vs. 80.7 pg/mL; *P* = 0.0499) and leptin levels (median; 16,081 vs. 6770 pg/mL; *P* = 0.0224), but not those with MOGAD. Better improvement in EDSS at 6 months was associated with baseline BMP-9 levels in patients with AQP4 + NMOSD (Spearman’s rho =  − 0.47; *P* = 0.037). Serum BMP-9 is upregulated at relapse and may contribute to vascular remodeling in AQP4 + NMOSD. Serum BMP-9 levels could predict clinical recovery 6 months after the attack.

## Introduction

Neuromyelitis optica spectrum disorder (NMOSD), an inflammatory disorder in the central nervous system (CNS), is considered a subtype of multiple sclerosis (MS), and NMO-IgG findings differentiated NMOSD from MS^1^. The positivity of anti-aquaporin-4 antibodies (AQP4-IgG) was one of the NMOSD features^2^, but recent studies reported some patients with AQP4-IgG-negative NMOSD having positive anti-myelin oligodendrocyte glycoprotein (MOG-IgG)^3,4^. Additionally, similar clinical features and laboratory findings, including increased cerebrospinal fluid (CSF) interleukin-6 (IL-6) levels, were reported in AQP4-IgG-positive NMOSD (AQP4 + NMOSD) and MOG-IgG-associated disorders (MOGAD)^5–7^. Favorable clinical outcomes were reported in MOGAD compared with AQP4 + NMOSD even in the first optic neuritis^8,9^. However, the mechanism behind milder clinical outcomes in MOGAD compared with AQP4 + NMOSD was not fully elucidated. A recent study reported the different patterns and extent of helper T cell profiles between AQP4 + NMOSD and MOGAD^10^. Therefore, AQP4 + NMOSD and MOGAD have distinct pathophysiological differences.

Meanwhile, the meningeal lymphatic vasculature regulated CNS lymphatic drainage and neuroinflammation^11^. Surgical and pharmacological blockade of lymphatic function attenuated experimental autoimmune encephalomyelitis (EAE), which is the animal model of MS. Inflammation has induced neuro-lymphatic protein expression in MS brain vasculature^12^. Recurrent inflammation regulated lymphatic vessel remodeling^13^, and vascular remodeling is relevant to blood–brain barrier (BBB) remodeling. Therefore, we hypothesized that cytokines relevant to vascular regeneration as a short-term prognostic factor after attacks in AQP4 + NMOSD. This study investigated vascular regeneration cytokine profiles after attacks and their prognostic factor in AQP4 + NMOSD.

## Materials and methods

### Standard protocol approvals and patient consent

The study procedure was approved by the ethics committee of the Chiba University School of Medicine (Nos. 842 and 1937) and Sannou Hospital. All patients provided written informed consent. The methods used in this study comply with the Declaration of Helsinki and its subsequent amendments, and were performed in accordance with the relevant guidelines and regulations.

### Participants and samples

This study recruited 22 patients with AQP4 + NMOSD, 20 healthy controls (HCs), and 20 patients with MOGAD as disease controls. All patients with AQP4 + NMOSD fulfilled the 2015 international diagnostic criteria for NMOSD^2^. Anti-MOG-IgG-positive disorders with CNS involvement were defined as MOGAD^10^. The presence of AQP4-IgG and MOG-IgG antibodies was confirmed by a cell-based assay measured as previously described^14^.

Serum samples of HCs were obtained as previously reported. Briefly, 196 HCs who underwent a complete medical check-up at Sannou Hospital provided serum samples as volunteers^15,16^, of whom 20 age- and sex-matched samples with MOGAD were included in the study.

Serum and CSF samples in the acute phase were obtained before giving any attack treatment, including steroids and plasma exchange, in patients with AQP4 + NMOSD and MOGAD.

Demographic characteristics, including sex ratio and age at sampling, and clinical features, including disease duration to sampling, the percentage of the first attack, and Kurtzke’s Expanded Disability Status Scale (EDSS) before the attack, at sampling, and 6 months after the attack, were investigated. The prognostic factor at 6 months after the attack was analyzed by investigating the correlation between ΔEDSS (6 M-pre), which is EDSS at 6 months minus EDSS before the attack, and clinical items or cytokine levels in the acute phase. Laboratory findings, including CSF cell count, CSF protein concentration, the quotient of albumin (Qalb), IgG index, and the percentage with positive oligoclonal IgG bands, and baseline treatment at sampling and treatment in the acute phase were compared. The effects of treatments on ΔEDSS (6 M-pre) or cytokine levels relevant to better improvement in EDSS at 6 months after the attack were also investigated.

### Cytokine measurements

All serum samples, just after centrifugation at 3000 rpm for 10 min, and all CSF samples were immediately stored at − 80 °C until cytokine analysis, other than IL-6. The serum and CSF IL-6 levels were measured on a single detection immediately after centrifugation at room temperature using the electrochemiluminescence immunoassay according to the manufacturer’s instruction (Roche Diagnostics K.K., Tokyo, Japan). The serum cytokine concentrations relevant to the blood vessel regeneration were measured using the MILLIPLEX® (Merck Millipore, Darmstadt, Germany) human angiogenesis/growth factor magnetic bead panel 1 with a single detection, according to the manufacturer's instruction. Fluorescence intensity from the immunoassay was acquired and analyzed using xPONENT 4.2 Software (Luminex Corporation, Austin, TX, USA). The measured cytokines relevant to the vascular remodeling included epidermal growth factor, angiopoietin 2, granulocyte-colony stimulating factor (G-CSF), bone morphogenetic protein-9 (BMP-9), endoglin, leptin, hepatocyte growth factor (HGF), placental growth factor, vascular endothelial growth factor (VEGF)-C, VEGF-D, fibroblast growth factor-2 (FGF-2), and VEGF-A. Values under the dynamic range were replaced by half of the lower sensitivity limit.

### Statistical analysis

JMP pro version 15.0.0 (SAS Institute Inc, Cary, NC, USA) was used for statistical analysis. Continuous data were compared using the Mann–Whitney *U* test or Steel test HCs as control. The Steel–Dwass test was performed on the items with statistical differences. Categorical outcomes were evaluated using Fisher’s exact test. Spearman’s rank test was performed to analyze correlations between elevated cytokines compared with HCs and clinical items. Correlations between clinical items and serum or CSF IL-6 levels were investigated by Spearman’s rank test. The *p-*value was considered significant at 0.05. Since prednisolone could affect the cytokine levels including BMP-9^17^, an analysis of covariance (ANCOVA) was added to evaluate the effect of prednisolone on the cytokines which were shown to be related to prognosis using significant different items with or without prednisolone as covariates.

### Ehical approval

The study procedure was approved by the ethics committee of the Chiba University School of Medicine (No. 842 and 1937) and Sannou Hospital.

### Informed consent

All patients provided written informed consent.

## Results

### Demographics and clinical characteristics in patients and HCs

Table 1 shows the demographic and clinical characteristics of patients. This study excluded 2 patients with AQP4 + NMOSD, 4 with MOGAD, and 3 HCs because of the shortage of included beads for measuring cytokines. Finally, 20 patients with AQP4 + NMOSD, 18 patients with MOGAD, and 17 HCs were included.

**Table 1:** The demographic and clinical characteristics of patients with AQP4 + NMOSD, MOGAD, and HCs.

	AQP4 + NMOSD (N = 20)	MOGAD (N = 18)	HCs (N = 17)	*P* value
				AQP4 + NMOSD vs MOGAD
Demographic and clinical features				
Female (%)	17/20 (85.0)	12/18 (66.7)	13/17 (76.5)	0.2603
Age (years)	51.5 (15.3)	45.0 (37.5)	44.0 (6.0)	0.7536
Disease duration (years)	2.0 (5.5)	0.0 (1.8)		0.0220
Days from attack to sampling in acute phase	14.5 (17.5)	14.0 (18.3)		0.8262
Days from attack to treatment in acute phase	15.0 (17.0)	12.5 (19.0)		0.6666
EDSS before the attack	1.5 (2.0)	0.0 (1.0)		0.0324*
EDSS at sampling in acute phase	4.0 (2.8)	5.0 (3.0)		0.5837
EDSS at 6 months after attack	2.0 (3.3)	1.0 (2.0)		0.0013*
ΔEDSS (6 M-pre)	0.75 (2.4)	0.0 (1.5)		0.2406
First attack (%)	4/20 (20.0)	11/18 (61.1)		0.0189*
Laboratory findings				
CSF cell count (/μL)	5.8 (10.0)	3.2 (8.0)		0.1784
CSF protein concentration (mg/dl)	44.5 (18.8)	32.0 (38.5)		0.2852
Qalb (*10^–3^)	5.8 (3.0)	4.3 (5.2)		0.3495
IgG index	0.67 (0.19)	0.62 (0.19)		0.2360
Positive oligoclonal IgG bands (%)	6/16 (37.5)	2/18 (11.1)		0.1131
Baseline treatments				
Prednisolone only	10	3		
Prednisolone plus immunosuppressant	1	1		
Prednisolone plus regular plasma exchange	1	0		
None	8	14		
Treatments in the acute phase				
mPSL pulse	11	12		
mPSL pulse plus plasma exchange	8	4		
Oral prednisolone	0	0		
None	1	2		

Data are presented as median [interquartile range] or number (%). *P < 0.05.

ΔEDSS (6 M-pre) = EDSS at six months after the attack minus EDSS before the attack.

Immunosuppressant includes azathioprine and tacrolimus.

AQP4 + NMOSD: anti-aquaporin-4-IgG positive neuromyelitis optica, CSF: cerebrospinal fluid, EDSS: Kurtzke’s Expanded Disability Status Scale, MOGAD: anti-myelin oligodendrocyte glycoprotein IgG associated disorders, mPSL: methyl prednisolone, Qalb: quotient albumin.

The female-to-male ratio was not different among the three groups. The percentages of the female were 85.0, 66.7, and 76.5% for AQP4 + NMOSD, MOGAD, and HCs, respectively. The median age was 51.5, 45.0, and 44.0 for AQP4 + NMOSD, MOGAD, and HCs, respectively (interquartile range; 15.3, 37.5, and 6.0, respectively). Ages were not different among AQP4 + NMOSD, MOGAD, and HCs, and disease duration was not different between AQP4 + NMOSD and MOGAD. EDSS before the attack and at 6 months was higher in patients with AQP4 + NMOSD compared to those with MOGAD (median; 1.5 vs. 0.0 and 2.0 vs. 1.9, *P* = 0.0324 and 0.0013, respectively). The percentage of patients with the first attack was higher in patients with MOGAD (61.1%) than in patients with AQP4 + NMOSD (61.1% vs. 20.0%, respectively, *P* < 0.001). Laboratory findings revealed no difference between CSF cell count, CSF protein concentration, Qalb, IgG index, and oligoclonal IgG bands positivity and AQP4 + NMOSD and MOGAD.

The median days from attack to sampling in the acute phase were 14.5 and 14.0 in patients with AQP4 + NMOSD and MOGAD, respectively (interquartile range: 17.5 and 18.3, range: 2–68 days and 1–81 days, respectively).

### Cytokine profiles in patients with AQP4 + NMOSD and MOGAD in the acute phase and HCs

**Figure 1:**
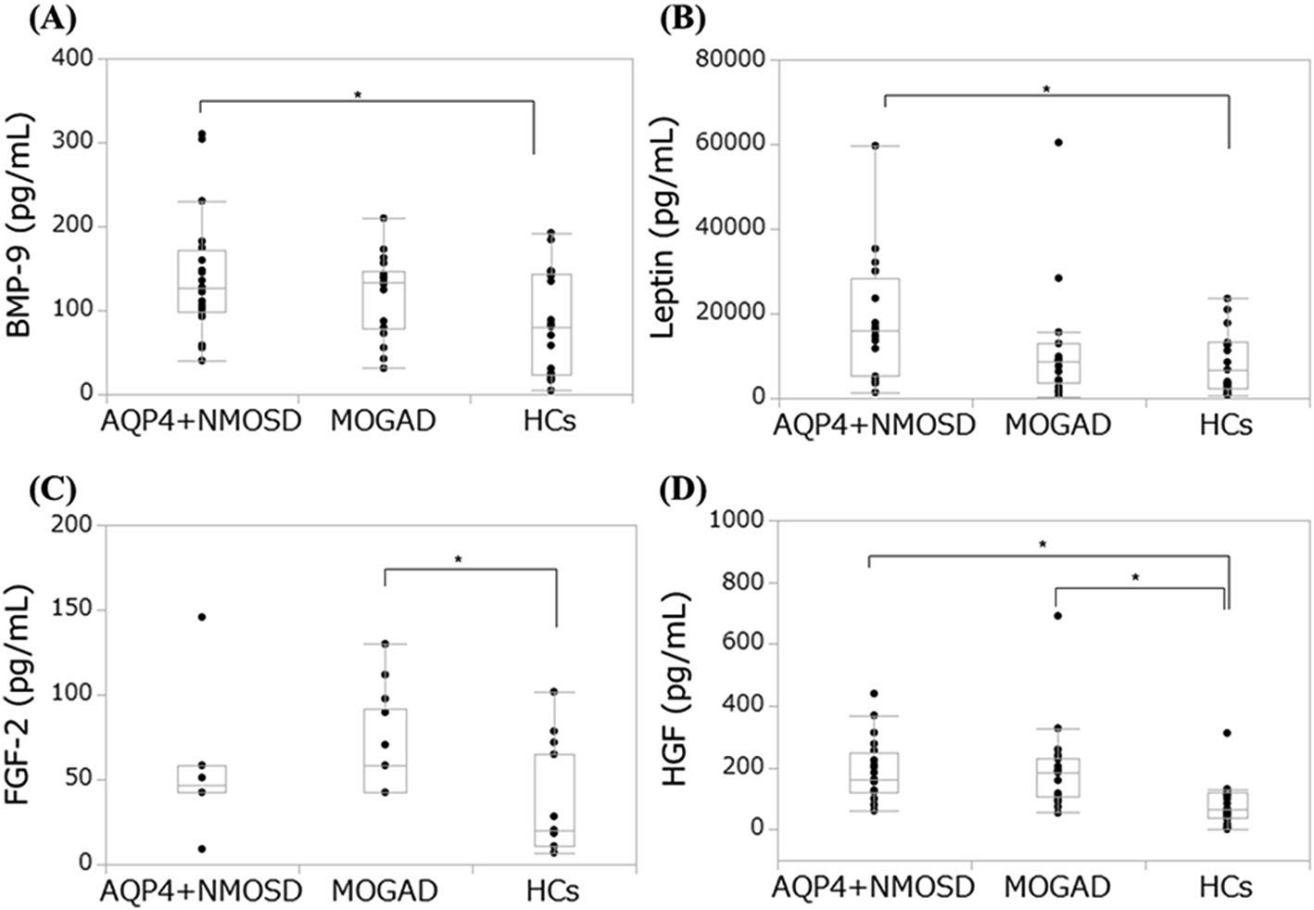
Elevated cytokine levels in AQP4 + NMOSD, MOGAD, and HCs. Box plots are demonstrated for each group. *Statistically significant after the Steel test HCs as control. AQP4 + NMOSD: anti-aquaporin-4-IgG positive neuromyelitis optica spectrum disorder, HCs: healthy controls, MOGAD: anti-myelin oligodendrocyte glycoprotein IgG associated disorders.

Table 2 shows the cytokine profiles in patients with AQP4 + NMOSD and MOGAD in the acute phase and those in HCs. Patients with AQP4 + NMOSD showed higher BMP-9 and leptin compared with HCs. FGF-2 was elevated in patients with MOGAD compared with HCs. HGF levels were increased in AQP4 + NMOSD and MOGAD. Figure 1 shows the elevated cytokines compared with HCs**.** Leptin levels in patients with AQP4 + NMOSD remained higher compared with HCs after the Steel–Dwass test for BMP-9 and leptin (*P* = 0.0224). HGF was higher in AQP4 + NMOSD and MOGAD compared with HCs after the Steel–Dwass test (*P* = 0.0006 and 0.0033, respectively). Vascular regeneration-related cytokine and IL-6 levels were not different between AQP4 + NMOSD and MOGAD.

**Table 2:** Cytokine profile in patients with AQP4 + NMOSD and MOGAD in the acute phase and those in HCs.

	HCs (N = 17)	AQP4 + NMOSD (N = 20)	MOGAD (N = 18)	*P* value		
				AQP4 + NMOSD vs HCs	MOGAD vs HCs	AQP4 + NMOSD vs MOGAD
**Serum**						
IL-6 (pg/mL)	N.A	3.2 (4.3) (N = 15)	1.7 (2.3) (N = 14)			0.2171
EGF (pg/mL)	24.9 (82.6)	25.9 (58.5)	39.7 (61.8)	0.9335	0.7422	
Angiopoietin 2 (pg/mL)	833 (1169)	807 (549)	543 (572)	0.9792	0.2861	
G-CSF (pg/mL)	13.7 (45.7)	20.8 (17.8)	18.5 (11.6)	0.7918	0.2688	
BMP-9 (pg/mL)	80.7 (119)	127 (71.7)	134 (68.9)	0.0499*	0.2273	
Endoglin (pg/mL)	936 (524)	993 (359)	1094 (267)	0.5449	0.2706	
Leptin (pg/mL)	6770 (10,859)	16,081 (23,260)	8829 (9188)	0.0224*	0.8989	
HGF (pg/mL)	66.7 (81.6)	163 (125)	186 (127)	< 0.001*	0.0022*	
PLGF (pg/mL)	2.2 (4.5)	3.9 (4.2)	3.0 (3.8)	0.5033	0.9430	
VEGF-C (pg/mL)	836 (1447)	513 (913)	1305 (1053)	0.9129	0.1776	
VEGF-D (pg/mL)	117 (655)	55.0 (46.7)	59.1 (56.0)	0.0836	0.1234	
FGF-2 (pg/mL)	20.3 (54.2)	46.8 (15.9)	58.4 (49.1)	0.0779	0.0070*	
VEGF-A (pg/mL)	175 (179)	274 (418)	259 (194)	0.1236	0.6048	
CSF						
IL-6 (pg/mL)	N.A	12.4 (16.8) (N = 16)	9.9 (22.6) (N = 17)			0.8009

Data are presented as median [interquartile range]. *P < 0.05.

AQP4 + NMOSD: anti-aquaporin-4-IgG positive neuromyelitis optica spectrum disorder, BMP-9: bone morphogenetic protein-9, CSF: cerebrospinal fluid, EGF: epidermal growth factor, FGF-2: fibroblast growth factor-2, G-CSF: granulocyte-colony stimulating factor, HCs: healthy controls, HGF: hepatocyte growth factor, IL-6: interleukin-6, MOGAD: anti-myelin oligodendrocyte glycoprotein IgG associated disorders, N.A.: not acquired, PLGF: placental growth factor, VEGF: vascular endothelial growth factor.

### Correlations between elevated cytokines and clinical items or IL-6 levels in patients with AQP4 + NMOSD

**Figure 2:**
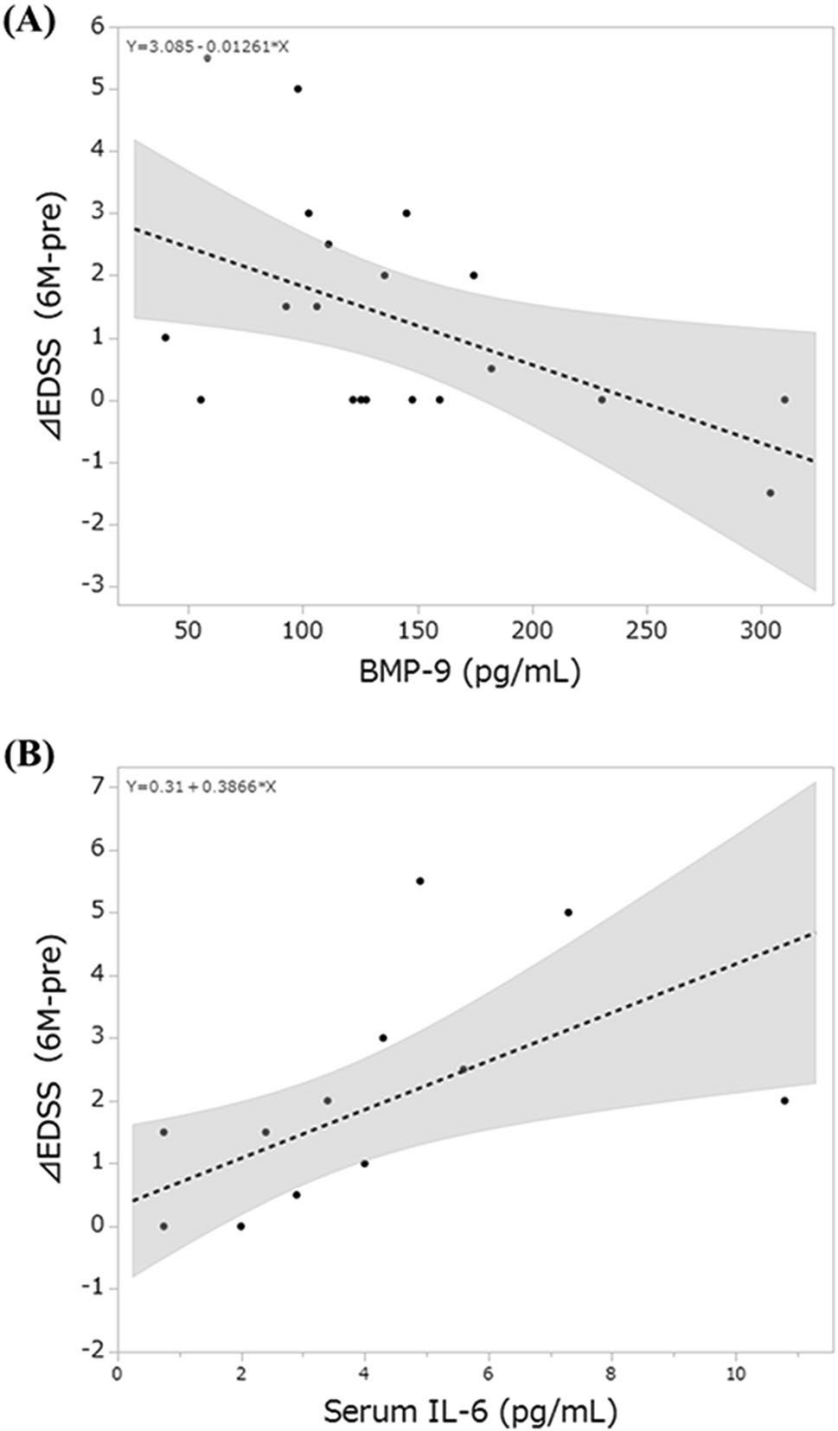
Correlations between elevated cytokine levels compared with HCs and clinical items in patients with AQP4 + NMOSD. (A) Negative correlation between ΔEDSS (6 M-pre) and BMP-9. (B) Positive correlation between ΔEDSS (6 M-pre) and serum IL-6 levels. AQP4 + NMOSD: anti-aquaporin-4-IgG positive neuromyelitis optica spectrum disorders, CSF: cerebrospinal fluid, BMP-9: bone morphogenetic protein-9, EDSS; Kurtzke’s Expanded Disability Status Scale, IL-6: interleukin-6. ΔEDSS (6 M-pre) = EDSS at 6 months after the attack minus EDSS before the attack.

Table 3 shows the correlations between elevated cytokines compared with HCs and clinical items. Positive correlations were found between BMP-9 levels and disease duration or EDSS in patients with AQP4 + NMOSD before the attack. Conversely, BMP-9 levels negatively correlated with ΔEDSS (6 M-pre) (rho =  − 0.4690, *P* = 0.0370, Fig. 2A). BMP-9 levels showed no correlations with other clinical items, including age, CSF cell count, CSF protein, Qalb, IgG index, EDSS at sampling, and EDSS 6 months after the attack. BMP-9 levels suggested the negative correlation with CSF IL-6 levels (rho =  − 0.4823, *P* = 0.0567) and exhibited no correlation with serum IL-6 levels (*P* = 0.7277). Meanwhile, leptin levels positively correlated with Qalb (rho = 0.4586, *P* = 0.0420). No correlations were found between leptin levels and other clinical items, including age, disease duration, CSF cell count, CSF protein, Qalb, IgG index, EDSS before the attack, EDSS at sampling, EDSS 6 months after the attack, and ΔEDSS (6 M-pre). HGF positively correlated with disease duration (rho = 0.4110, *P* = 0.0104), EDSS before the attack (rho = 0.4014, *P* = 0.0125), and EDSS 6 months after the attack (rho = 0.3834, *P* = 0.0175). HGF levels showed no correlations with serum or CSF IL-6 levels.

**Table 3:** Spearman’s rank correlation coefficient (rho) of the correlation between elevated cytokines or IL-6 levels and clinical items in AQP4 + NMOSD and MOGAD.

	Age	Disease duration	CSF cell count	CSF protein	Qalb	IgG index	EDSS before attack	EDSS in the acute phase	EDSS six months after attack	ΔEDSS (6 M-pre)
AQP4 + NMOSD										
BMP-9 (pg/mL)	0.1387 (*P* = 0.5599)	0.5444* (*P* = 0.0131)	− 0.4197 (*P* = 0.0654)	0.1911 (*P* = 0.4196)	0.2316 (*P* = 0.3259)	− 0.4228 (*P* = 0.0713)	0.4927* (*P* = 0.0273)	− 0.1667 (*P* = 0.4825)	0.1507 (*P* = 0.5260)	− 0.4690* (*P* = 0.0370)
Leptin (pg/mL)	0.0256 (*P* = 0.9146)	0.1575 (*P* = 0.5072)	0.2231 (*P* = 0.3445)	0.4266 (*P* = 0.0607)	0.4586* (*P* = 0.0420)	− 0.1526 (*P* = 0.5328)	0.2274 (*P* = 0.3349)	− 0.2546 (*P* = 0.2788)	0.2295 (*P* = 0.3304)	− 0.0894 (*P* = 0.7077)
HGF (pg/mL)	0.1713 (*P* = 2112)	0.4110* (*P* = 0.0104)	0.0085 (*P* = 0.9597)	0.2169 (*P* = 0.1909)	0.2968 (*P* = 0.0704)	− 0.0567 (*P* = 0.7389)	0.4014* (*P* = 0.0125)	0.0600 (*P* = 0.7204)	0.3834* (*P* = 0.0175)	0.1139 (*P* = 0.4959)
Serum IL-6 (pg/mL)	− 0.0585 *(P* = 0.8359)	− 0.4130 (*P* = 0.1260)	0.5553* (*P* = 0.0316)	0.0510 (*P* = 0.8569)	0.0946 (*P* = 0.7375)	− 0.1309 (*P* = 0.6419)	− 0.5078 (*P* = 0.0533)	0.36232 (*P* = 0.1844)	0.4605 (*P* = 0.0841)	0.8312* (*P* = 0.0001)
CSF IL-6 (pg/mL)	0.2762 (*P* = 0.3004)	− 0.0391 (*P* = 0.8856)	0.4709 (*P* = 0.0656)	0.3444 (*P* = 0.1915)	0.2559 (*P* = 0.3388)	0.5353* (*P* = 0.0326)	0.0923 (*P* = 0.7339)	0.5015* (*P* = 0.0478)	0.1665 (*P* = 0.5377)	0.1648 (*P* = 0.5418)
MOGAD										
HGF (pg/mL)	0.0884 (*P* = 0.7272)	0.5410 (*P* = 0.0204)	− 0.0451 (*P* = 0.8590)	0.0415 (*P* = 0.8703)	0.0950 (*P* = 0.7076)	0.1632 (*P* = 0.5175)	0.5234* (*P* = 0.0258)	− 0.0550 (*P* = 0.8284)	0.4347 (*P* = 0.0715)	0.1883 (*P* = 0.4544)
FGF-2 (pg/mL)	− 0.2444 (*P* = 0.3283)	0.1655 (*P* = 0.5116)	− 0.3588 (*P* = 0.1437)	− 0.0842 (*P* = 0.7399)	0.0290 (*P* = 0.9089)	− 0.3926 (*P* = 0.1071)	0.3018 (*P* = 0.2236)	− 0.2735 (*P* = 0.2721)	0.3644 (*P* = 0.1371)	0.2646 (*P* = 0.2886)
Serum IL-6 (pg/mL)	0.1456 (*P* = 0.6195)	− 0.1377 (*P* = 0.6387)	0.1469 (*P* = 0.6163)	− 0.2949 (*P* = 0.3060)	− 0.2974 (*P* = 0.3018)	0.3477 (*P* = 0.2232)	− 0.5359* (*P* = 0.0482)	− 0.0657 (*P* = 0.8235)	− 0.0159 (*P* = 0.9569)	0.2269 (*P* = 0.4352)
CSF IL-6 (pg/mL)	0.1141 (*P* = 0.6628)	0.0618 (*P* = 0.8138)	0.3567 (*P* = 0.1599)	0.3247 (*P* = 0.2035)	0.3186 (*P* = 0.2126)	0.4779 (*P* = 0.0523)	0.1400 (*P* = 0.5919)	0.3397 (*P* = 0.1822)	0.6799* (*P* = 0.0027)	0.7211* (*P* = 0.0011)

Data are presented as median [interquartile range]. *P < 0.05.

AQP4 + NMOSD: anti-aquaporin-4-IgG positive neuromyelitis optica spectrum disorder, BMP-9: bone morphogenetic protein-9, CSF: cerebrospinal fluid, FGF-2: fibroblast growth factor-2, HGF: hepatocyte growth factor, IL-6: interleukin-6, MOGAD: anti-myelin oligodendrocyte glycoprotein IgG associated disorders.

### Treatment effects on ΔEDSS (6 M-pre) and BMP-9 levels in patients with AQP4 + NMOSD

In patients with AQP4 + NMOSD, ΔEDSS (6 M-pre) and BMP-9 levels showed no difference between methylprednisolone pulse therapy and methylprednisolone pulse plus plasma exchange in the acute phase (*P* = 0.3449 and 0.4828, respectively). Patients with AQP4 + NMOSD who received only prednisolone as a baseline treatment showed lower ΔEDSS (6 M-pre) compared with those without baseline treatments at the attack (median; 0.0 vs 2.25, interquartile range; 1.25 vs 3.0, *P* = 0.0184). BMP-9 levels were not different between patients with AQP4 + NMOSD who received only prednisolone as a baseline treatment and those without baseline treatments (*P* = 0.0832).

### BMP-9 levels were not different with or without prednisolone after ANCOVA in patients with AQP4 + NMOSD

To exclude the prednisolone effects on BMP-9 expression, the demographic and clinical characteristics were compared with or without prednisolone in patients with AQP4 + NMOSD. The results showed the longer disease duration (median; 5.0 years vs 0.5 years, interquartile range; 11.3 vs 1.0, *P* = 0.0206) and days from attack to sampling in acute phase (median; 7.0 vs 27.0, interquartile range; 14.5 vs 30.8, *P* = 0.0339) in patients with prednisolone compared with those without prednisolone. Age, sex, days from attack to treatment in the acute phase, EDSS before the attack, and EDSS at the sampling in the acute phase were not different between the two groups. ANCOVA showed no difference in BMP-9 levels with or without prednisolone when disease duration and days from attack to sampling in the acute phase were used as covariates (*P* = 0.151).

### Correlations between elevated cytokines and clinical items or IL-6 levels in patients with MOGAD

HGF showed the positive correlation with EDSS before the attack in patients with MOGAD (rho = 0.5234, *P* = 0.0258). No correlations were found between HGF levels and clinical items other than EDSS before the attack. FGF-2 demonstrated no correlations with all clinical items. Further, HGF and FGF-2 showed no correlation with serum or CSF IL-6 levels.

### Correlations between serum or CSF IL-6 levels and clinical items in patients with AQP4 + NMOSD and MOGAD

Serum IL-6 levels in patients with AQP4 + NMOSD showed the positive correlation with CSF cell count (rho = 0.5553, *P* = 0.0316) and ΔEDSS (6 M-pre) (rho = 0.8312, *P* = 0.0001, Fig. 2B). CSF IL-6 levels positively correlated with IgG index (rho = 0.5353, *P* = 0.0326) and EDSS at sampling in the acute phase (rho = 0.5015, *P* = 0.0478). Meanwhile, CSF IL-6 levels in patients with MOGAD positively correlated with ΔEDSS (6 M-pre) (rho = 0.7211, *P* = 0.0011) and EDSS 6 months after the attack (rho = 0.6799, *P* = 0.0027). Serum IL-6 levels negatively correlated with EDSS before the attack (rho =  − 0.5359, *P* = 0.0482).

### Serum IL-6 levels were not different with or without prednisolone after ANCOVA in patients with AQP4 + NMOSD

No statistical difference was found in serum IL-6 levels with or without prednisolone in patients with AQP4 + NMOSD after ANCOVA was performed with disease duration and days from attack to sampling in the acute phase as covariates (*P* = 0.564).

## Discussion

This study revealed elevated BMP-9, leptin, and HGF levels in patients with AQP4+NMOSD and FGF-2 and HGF levels in patients with MOGAD in the acute phase compared with HCs. ΔEDSS (6M-pre) in patients with AQP4+NMOSD showed a negative correlation with BMP-9 levels and a positive correlation with serum IL-6 levels. Leptin levels positively correlated with Qalb in patients with AQP4+NMOSD. Lower ΔEDSS (6M-pre) was observed in patients with AQP4+NMOSD who received only prednisolone as a baseline treatment compared with those without baseline treatments.

BMP-9 is produced by hepatic stellate cells in the liver and is a differentiating factor for cholinergic CNS neurons^18,19^. Additionally, BMP-9 prevents vascular permeability by VEGF-receptor-2 signaling and vascular endothelial (VE)-cadherin internalization and occludin expression promotion^18^. BMP-9 administration in neurological diseases improves memory and alleviates the pathology in the animal model of Alzheimer’s disease^20,21^. BMP-9 overexpression decreases cell death and improves cell viability in astrocytes in the cerebral ischemia–reperfusion rat model^22^. AQP4-IgG was reported to target astrocytes^2,23,24^. Severe astrocytic damage in neuromyelitis optoca was also demonstrated^25^. Therefore, decreasing astrocyte death by BMP-9 overexpression could lead to improve EDSS 6 months after the attack in patients with AQP4 + NMOSD. Our study indicates higher serum BMP-9 in the acute phase as a good prognostic factor to predict disability severity 6 months after the attack in patients with AQP4 + NMOSD. To our best knowledge, no other study about BMP-9 was performed in the field of AQP4 + NMOSD.

The results demonstrated a positive correlation between serum IL-6 levels in patients with AQP4 + NMOSD and ΔEDSS (6 M-pre). CSF IL-6 levels were negatively correlated with BMP-9 levels in patients with AQP4 + NMOSD. IL-6 plays an important role in NMOSD pathogenesis^5,26,27^. Increased serum and CSF IL-6 levels were demonstrated in the acute phase in patients with NMOSD^6^. Additionally, CSF IL-6 levels were elevated in the acute phase in patients with NMOSD and MOGAD^7^. Two recent randomized controlled trials confirmed that IL-6 receptor inhibition reduced NMOSD relapses^28,29^. Hence, our study suggests serum IL-6 levels in patients with AQP4 + NMOSD as a short-term prognostic factor in those diseases.

Our study revealed elevated leptin levels and a positive correlation between leptin levels and Qalb in patients with AQP4 + NMOSD. A previous study reported increased serum leptin levels in patients with NMO and MS^30^. Leptin was reported to protect the brain from ischemic injury by reducing neuronal cell death^31–33^. A recent study revealed that leptin protected the brain from ischemia by stabilizing the BBB^34^. Therefore, our result may be explained by the positive feedback of leptin in stabilizing the BBB in the acute AQP4 + NMOSD phase.

Our study revealed higher HGF levels in patients with AQP4 + NMOSD and MOGAD compared with HCs. Previous studies reported that HGF alleviates EAE severity and limits cytotoxic T-cell generation and its effector functionsyy^35,36^. However, our study revealed no correlation between HGF and ΔEDSS (6 M-pre). To our best knowledge, this is the first report to show HGF elevation in patients with AQP4 + NMOSD. Therefore, further investigation is required to conclude HGF function in AQP4 + NMOSD pathogenesis.

Our study has some limitations. First, the percentage of the first attack was higher in patients with MOGAD compared to those with AQP4 + NMOSD, which could lead to baseline treatment differences, thereby affecting the cytokine values, particularly in the acute phase in patients with AQP4 + NMOSD. Second, body mass index was not obtained in our study. As leptin is associated with adipose tisse, difference in body mass index among the groups could affect the results. Finally, each group had a small sample size. Hence, a prospective study with increased samples and adjusted baseline treatment is required in the future.

In conclusion, our study demonstrated that serum BMP-9 levels could predict clinical recovery 6 months after an attack in patients with AQP4 + NMOSD. However, prospective studies with larger sample size are required to confirm our results.

## Data Availability

The data that support our findings are available from the corresponding author, upon reasonable request.
